# Availability of drugs for severe COVID-19 in sub-Saharan Africa

**DOI:** 10.11604/pamj.supp.2020.35.2.23698

**Published:** 2020-05-26

**Authors:** Sylvain Raoul Simeni Njonnou, Christian Ngongang Ouankou, Fernando Kemta Lekpa, Eric Vounsia Balti, Simeon Pierre Choukem

**Affiliations:** 1 Department of Internal Medicine and Specialties, Faculty of Medicine and Pharmaceutical Sciences, University of Dschang, Dschang, Cameroon; 2 The University of Dschang Taskforce for the Elimination of COVID-19 (UNITED#COVID-19**), Dschang, Cameroon; 3 Department of Internal Medicine and Specialties, Yaoundé University Teaching Hospital, Yaoundé, Cameroon; 4 Department of Internal Medicine, Douala General Hospital, Douala, Cameroon; 5 Diabetes Research Center and Department of Internal Medicine, Universiteit Ziekenhuis Brussel, Vrije Universiteit Brussel, Brussels, Belgium; 6 Health and Human Development (2HD) Research Network, Douala, Cameroon

**Keywords:** COVID-19, effective treatment, severe, drug availability

## To the editors of the Pan African Medical Journal

The current outbreak of the new coronavirus disease (COVID-19), caused by the Severe Acute Respiratory Syndrome Coronavirus-2 (SARS-CoV-2), initially originating from Wuhan, in China, has nowadays spread to all the continents. This pandemic leads to great challenges and sets a major burden on health systems. One of these challenges is the development of effective treatments, particularly for severely ill patients with COVID-19. Sanders et al. described several potential treatment targets, based on the current understanding of the pathophysiology of this disease [1]. Among the drugs targeting these processes, some have been rather disappointing. The association lopinavir-ritonavir has not shown any benefit in severe COVID-19 so far [2]. The initial worldwide enthusiasm for the use of hydroxychloroquine (associated or not with azithromycin) [3] seems to give way in the light of the latest evidence to an increase in the risk of ventricular arrhythmia [4]. Current data on tocilizumab and remdesivir, from case series and randomized controlled trials, show interesting results in severe and critical COVID-19 [5-7]. Tocilizumab is a recombinant humanized monoclonal antibody that binds to the interleukin 6 (IL-6) receptor. The use of this treatment option relies on the pathophysiology of severe COVID-19 which is closely linked to a cytokine storm in which IL-6 plays a major role.

In a recent report, Xu et al. describe a series of severely and critically ill patients with COVID-19 treated with tocilizumab in two reference hospitals of China [7]. Disease severity was assessed using high respiratory rate (≥30 breaths/minute), decreased capillary oxygen saturation at room air (SpO2 ≤ 93%) and compliance with criteria of acute respiratory distress syndrome based on PaO2/FiO2 ≤ 300 mm Hg. Critical cases were reported as such when respiratory failure required ventilation support and/or in the presence of features of shock with or without failure of other organs that needed admission to the intensive care unit. The use of tocilizumab in severely or critically ill COVID-19 patients led to a reduction of invasive ventilation needs as well as oxygen support, decline in C-reactive protein values, increase in the percentage of lymphocytes in peripheral blood and improvement in radiological images by day five of treatment. Interestingly, there were no adverse effects accounted for, by the treatment [7]. Despite these promising results, as acknowledged by the authors, one needs to mention the limited sample size and the need for confirmatory results from an independent study population and further exploration in randomized controlled trials.

While looking at the panel of currently investigated therapies, we can´t refrain from having concerns for countries of sub-Saharan Africa (SSA) once effective treatment options will be confirmed and recommended. Indeed, healthcare systems in SSA settings are poorly prepared for the COVID-19 pandemic. The lack of sufficiently equipped critical care units, in addition to the absence of universal health coverage, increase patients´ vulnerability [8]. As a result, more than in other contexts, these health systems are and will be facing more difficulties in the management of patients with COVID-19. On the one hand, the expected high cost of both supportive and disease-specific treatments represents an additional challenge to the accessibility to the latter. On the other hand, the populations are already facing the burden of other communicable as well as non-communicable diseases, with rather limited resources. There is therefore an urgent need to anticipate finding solutions to cover expenditures related to the management of COVID-19 in SSA. Some solutions that could be developed to allow the availability of these drugs might include a partial waiver of the medications´ cost by pharmaceutical companies. Moreover, the out-of-pocket expenditure for patients could be reduced by governmental subsidies. The development of biosimilars would be an interesting alternative with costs reduced by around 30% [9]. However, the implementation of these tentative solutions will require concertation between various stakeholders including health care providers, patients´ associations, insurance companies, governments´ representatives, charity organizations, and pharmaceutical companies. The use of convalescent plasma could be a rescue solution using the existing blood bank facilities, where available, under strict regulation [10]. Well-conducted clinical trials on their efficacy and safety are however still warranted. Local solutions to identify alternative treatment options from traditional pharmacopeia should also be considered. However, one could not emphasize enough that all the stages of drug development -except in the context of drug repurposing- and marketing authorization should be completed before their use for COVID-19.

## Conclusion

The COVID-19 pandemic has generated various attempts to identify successful treatments specifically for severe cases. Current data suggest promising results using IL-6 receptor targeting therapy. However, questions on the accessibility of this treatment option, and others, in resource-limited settings including SSA should be anticipated. Addressing this concern will require the implication of various stakeholders in designing a sound health policy. Accessibility to medical supplies and attempts to lower treatment costs could help to overcome the pitfalls related to the management of severely ill patients.

## Competing interests

The author declares no competing interests.
